# A Multiresolution Breast Cancer CIBERSORTx Resource Validated for Accuracy, Interpretive Limits, and Biological and Clinical Coherence in Tumor Microenvironment Deconvolution

**DOI:** 10.3390/mps9030088

**Published:** 2026-06-02

**Authors:** Toru Hanamura, Akinori Takase, Masanori Oshi, Naoki Niikura

**Affiliations:** 1 Department of Breast Oncology, Tokai University School of Medicine, 143 Shimokasuya, Isehara 259-1193, Kanagawa, Japan; 2 Department of Life Science Support, Research Innovation Center, University Hospitals Sector, Tokai University, Isehara 259-1193, Kanagawa, Japan; 3 Department of Breast Surgery, Yokohama City University Hospital, 3-9 Fukuura, Kanazawa-ku, Yokohama 236-0004, Kanagawa, Japan

**Keywords:** breast cancer, tumor microenvironment, deconvolution, CIBERSORTx, scRNA-seq

## Abstract

Accurate deconvolution of bulk transcriptomes is essential for characterizing the breast cancer tumor microenvironment (TME), yet existing reference matrices incompletely capture tumor-specific cellular diversity. Here, we developed breast cancer–specific multiresolution CIBERSORTx signature matrices from single-cell RNA sequencing data and systematically evaluated their analytical performance and interpretability. Major-, minor-, and subset-level matrices were constructed and assessed using pseudo-bulk mixtures and pure cell profiles, while biological and clinical coherence were evaluated in TCGA-BRCA and the I-SPY2 cohort. All matrices demonstrated high accuracy in reconstructing pseudo-bulk compositions, with performance declining at finer resolution. Spillover increased with granularity but was largely restricted within related lineages. Lineage-wise deconvolution modestly reduced spillover but consistently decreased accuracy, highlighting the importance of cross-lineage transcriptional contrast. In external datasets, most inferred cell populations showed biologically coherent associations with canonical markers and pathways, whereas some fine-resolution subsets exhibited non-canonical patterns, likely reflecting intra-lineage trade-offs or context-dependent transcriptional states. In the I-SPY2 cohort, plasmablasts and selected myeloid populations were positively associated with pathological complete response, whereas fibroblastic and perivascular-like populations showed negative associations. These findings establish a validated and interpretable resource for breast cancer TME deconvolution and clarify its performance characteristics and limitations.

## 1. Introduction

Breast cancer is the most commonly diagnosed malignancy worldwide and the leading cause of cancer-related mortality in women [1]. Although recent advances in molecularly targeted therapies and immune checkpoint inhibitors have improved clinical outcomes, substantial biological heterogeneity persists, leading to wide variability in therapeutic responses [2]. Therefore, a deeper understanding of the underlying biology and factors governing treatment efficacy is critical for optimizing patient stratification and clinical decision making. The key contributors to this heterogeneity are the complex interactions among immune cells, fibroblasts, and vascular endothelial cells that collectively shape the tumor microenvironment (TME) [3,4].

The advent of single-cell RNA sequencing (scRNA-seq) has enabled the high-resolution characterization of the diverse cellular components of the breast cancer TME, and several studies have generated comprehensive atlases spanning the epithelial, immune, and stromal compartments [5,6,7,8]. These analyses identified breast-cancer-specific cell populations and subclusters, along with their potential roles in immune regulation and therapeutic responses. However, the application of scRNA-seq to large clinical cohorts remains limited by the high analytical costs and technical challenges associated with tissue dissociation and sample processing. Consequently, large breast cancer cohorts, such as The Cancer Genome Atlas (TCGA), METABRIC, and Sweden Cancerome Analysis Network—Breast [9,10,11,12,13] continue to serve as foundational resources for translational research and remain predominantly bulk-based, despite being established over a decade ago. Similarly, contemporary clinical trials, such as I-SPY2 [14], still rely primarily on bulk RNA-seq or microarray platforms for gene expression profiling, making the deconvolution of bulk transcriptomes an essential approach for estimating TME cellular composition.

CIBERSORTx is a widely used deconvolution algorithm that estimates the relative abundances of constituent cell types in bulk transcriptomes by regressing gene expression profiles against a reference matrix (referred to as a Signature Matrix in CIBERSORT/CIBERSORTx) [15]. However, the most commonly used reference matrices were generated from purified leukocytes [16] and therefore do not represent the full spectrum of stromal and non-hematopoietic populations present in the breast cancer TME. In addition, tumor-infiltrating immune cells undergo substantial transcriptional reprogramming, producing gene expression states that differ markedly from those of circulating leukocytes [17,18]. Consequently, reference profiles derived from blood-derived or pan-cancer datasets fail to capture the tumor-specific immune and stromal diversity characteristics of solid tumors, particularly in breast cancer. These limitations highlight the need for tissue- and disease-specific signature matrices that accurately reflect the cellular landscape of the breast cancer TME.

A growing number of studies have constructed tumor-derived scRNA-seq reference matrices and applied them to CIBERSORTx-based deconvolution of bulk transcriptomes [19,20,21]. However, the performance evaluations in these studies have largely focused on broad immune or stromal compartments, and the accuracy and spillover characteristics of fine-grained cellular subsets remain insufficiently defined. Although recent scRNA-seq studies have delineated increasingly fine cellular subsets that have been incorporated into deconvolution frameworks, performance assessment of reference matrices in such settings typically remains limited to coarse, high-level validations. Furthermore, even when fine-grained subsets are well-characterized at the single-cell level, their biological correspondence with bulk RNA-seq deconvolution has rarely been systematically examined. Consequently, when fine-resolution subsets are incorporated into signature matrices, users often lack practical guidance regarding their interpretability, biological significance, and potential analytical limitations, posing a major barrier to the broader application of scRNA-seq-derived matrices in real-world translational research.

These gaps highlight the need for a systematic, breast-cancer-specific evaluation of fine-resolution signature matrices, including their analytical accuracy, spillover behavior, and biological interpretability.

## 2. Methods

### 2.1. Data Sources and Preprocessing

scRNA-seq data from human breast cancers (100,000 cells from 26 primary tumors: 11 ER+, 5 HER2+, and 10 triple-negative breast cancers) were obtained from the Broad Institute Single Cell Portal (accession ID: SCP1039) as reported by Wu et al. (Nature Genetics, 2021 [8]). Bulk RNA-seq data for TCGA-Breast Invasive Carcinoma (BRCA) cohort (*n* = 1100) [9] were downloaded from Genomic Data Commons, converted to transcripts per million (TPM) using Seurat (v5.3.0) in R (v4.3.3), and mapped to official HUGO gene symbols using the biomaRt package (v2.58.2). Clinical data and log_2_-transformed microarray expression profiles from the pembrolizumab + paclitaxel arm of the I-SPY2 trial (*n* = 69) were obtained from the Gene Expression Omnibus (accession ID: GSE194040) [22].

### 2.2. Construction of Signature Matrix Files

In the original study by Wu et al. [8], breast cancer scRNA-seq data were clustered using Seurat and annotated based on canonical lineage markers and gene expression patterns. Each cell was annotated at three hierarchical levels, major, minor, and subset, as defined in the original study. Biological descriptions are available only for fine-grained subset-level clusters, which are summarized in Supporting Information S1; Supplementary Tables S1–S4. For matrix construction, 15% of the cells from each annotated category were randomly selected. This proportion was selected as a practical compromise to preserve representation of rare subsets while maintaining sufficient remaining cells for pseudo-bulk benchmarking and technical validation analyses. For cell types represented by fewer than 20 cells, the sample size was set to *n* = 20 to ensure adequate representation. Cell counts for all major, minor, and subset categories included in the reference matrices are provided in Supporting Information S1 and Supplementary Tables S5–S7. Raw counts were normalized to TPM and mapped to the official HUGO gene symbols, as described above. The minor and subset levels included several cycling populations derived from different lineages, such as cancer, perivascular-like (PVL), T-cell, and myeloid cycling. Because these groups contained few cells and their biological characteristics were insufficiently defined, they were consolidated into a single “Cycling” category and excluded from downstream analyses.

Signature matrices were generated in CIBERSORTx (https://cibersortx.stanford.edu/, accessed on 30 May 2025) using the “Create Signature Matrix” module, following the official user guide. Single-cell reference expression matrices were used as inputs, quantile normalization was disabled, and all other parameters were maintained at the default settings. Each matrix contained approximately 300–500 barcode genes. The resulting signature matrices and associated reference files were used for downstream deconvolution and batch correction analyses.

### 2.3. Validation of the Signature Matrix: Technical Validation

Technical validation was performed using pseudo-bulk mixtures and pure cell type samples generated from the same scRNA-seq dataset, excluding all cells used for matrix construction. Pseudo-bulk mixtures were created by first randomly generating target cell-type compositions, followed by random sampling of single cells according to the assigned proportions to generate mixtures containing 5000 aggregated cells. This process was repeated 100 times to generate diverse compositional states for benchmarking analyses. Pure cell type samples were constructed by aggregating the remaining cells from each annotated category. All mixtures were processed into TPM with HUGO gene symbol mapping, and deconvolution was performed in CIBERSORTx relative mode. The distribution of the true cell-type proportions at the major, minor, and subset levels is shown in Supporting Information S2; Supplementary Figure S1.

Deconvolution was performed in CIBERSORTx with S-mode batch correction, and B-mode and no-correction settings were evaluated for comparison. Quantile normalization was disabled and 100 permutations were performed. Accuracy was assessed primarily using Pearson’s correlation between the estimated and true proportions, with Spearman’s ρ and mean absolute error (MAE) as supplementary metrics. Spillover was evaluated using pure cell-type samples by comparing the estimated fractions with the expected values, and the results were visualized as heat maps showing diagonal accuracy and off-diagonal spillover.

Lineage-wise deconvolution was performed using a subset-level matrix subdivided into four lineages: cancer, stromal, lymphoid, and myeloid. Each lineage-specific matrix was applied independently using identical settings. Because CIBERSORTx outputs lineage-normalized fractions, the ground-truth proportions were recalculated on a lineage-restricted basis to enable a direct comparison. The accuracy and spillover were then assessed using the pseudo-bulk and pure cell-type samples described above.

### 2.4. Validation of the Signature Matrix: Biological Validation

The TCGA-BRCA bulk RNA-seq data were deconvoluted in CIBERSORTx (relative mode) using our breast cancer-specific signature matrices (major, minor, and subset levels) and the LM22 matrix, which was originally constructed from purified human leukocyte populations [16] for comparison. S-mode batch correction was applied to our matrices and B-mode was applied for LM22, with quantile normalization disabled, and 100 permutations performed. LM22-specific cell types that were not present in our matrices (mast cells, eosinophils, and neutrophils) were excluded. Biological consistency was assessed by correlating the estimated cell-type fractions with cluster-defining genes from Wu et al. (Supporting Information S1; Supplementary Tables S2–S4), supplemented with representative lineage markers from PanglaoDB [23].

Gene Set Variation Analysis (GSVA) was performed on TCGA-BRCA bulk transcriptomes using log_2_-transformed TPM values in the GSVA package (v2.4.1) in R v4.5.1. Hallmark gene sets were obtained from MSigDB, except that the canonical androgen response set was replaced with a breast cancer-specific AR signature, as previously described. GSVA was run with kcdf = “Gaussian” and a gene set size range of 10–5000 genes. To evaluate the biological plausibility of the breast-cancer-specific matrices, GSVA scores were correlated with the estimated cell-type fractions (minor and subset levels) using Pearson’s correlation. Correlation matrices were clustered hierarchically using 1—Pearson distance with average linkage. Hallmark pathways were ranked by maximal absolute correlation, and the lowest 20 were excluded from the analysis. The Myeloid_c5_Macrophage_3_SIGLEC1 subset was also removed because of its near-zero correlation with the true pseudo-bulk proportions. The resulting filtered correlation matrices were visualized as hierarchical heat maps.

Log_2_(TPM + 1) expression data from TCGA-BRCA were centered by subtracting the cohort-wide mean for each gene and were analyzed using the Tumor Immune Dysfunction and Exclusion (TIDE) framework [24]. From the TIDE outputs, T-cell dysfunction, M2-polarized tumor-associated macrophages (TAM M2), and cancer-associated fibroblast (CAF) scores were extracted as indicators of the TME state. Cytolytic activity (CYT) was calculated as the geometric mean of GZMA and PRF1 expression levels [25]. These indices were then correlated with the cell-type fractions estimated using our minor- and subset-level matrices, as well as LM22.

### 2.5. Clinical Validation in the I-SPY2 Trial

I-SPY2 transcriptome data were deconvoluted in CIBERSORTx (S-mode and relative mode) using our breast cancer-specific signature matrices. The resulting cell-type fractions were Z-transformed and analyzed using univariate logistic regression for pathological complete response (pCR) to estimate odds ratios and 95% confidence intervals. Because strong intercorrelations indicated multicollinearity, multivariable models were not fitted. Cell types with unreliable coefficient estimates or low deconvolution accuracy (e.g., Myeloid_c5_Macrophage _3_SIGLEC1) were excluded from the graphical presentation. In the minor and subset analyses, GM1–7 components were excluded because they reflect tumor-intrinsic programs rather than microenvironmental cell populations. All analyses were exploratory in nature.

### 2.6. Statistical Analysis

All statistical analyses and visualizations were performed using GraphPad Prism v10.6.1 (GraphPad Software, Boston, MA, USA), Jamovi v2.7.11.0 (The jamovi project, Sydney, Australia), and Morpheus (Broad Institute, Cambridge, MA, USA; https://software.broadinstitute.org/morpheus). Correlations between continuous variables were assessed primarily using Pearson’s r, with Spearman’s ρ computed for nonparametric validation. Paired two-group comparisons were conducted using the Wilcoxon matched-pairs signed-rank test, comparisons among three or more paired groups were performed using the Friedman test with Dunn’s post hoc test, and unpaired multi-group comparisons were evaluated using the Kruskal–Wallis test with Dunn’s post hoc test. Associations between deconvolved cell-type fractions and clinical outcomes were evaluated using logistic regression models. Odds ratios and 95% confidence intervals were calculated, with cell-type fractions treated as continuous variables. To account for multiple testing across cell types, *p*-values were adjusted using the Benjamini–Hochberg false discovery rate (FDR) method, and an FDR threshold of q < 0.1 was adopted given the exploratory nature of the analysis. All tests were two-sided. Statistical significance was defined as *p* < 0.05 for analyses not involving multiple testing, and as FDR q < 0.1 where multiple testing correction was applied.

## 3. Results

### 3.1. Evaluation of Deconvolution Accuracy Using Pseudo-Bulk Mixtures

**Figure 1:**
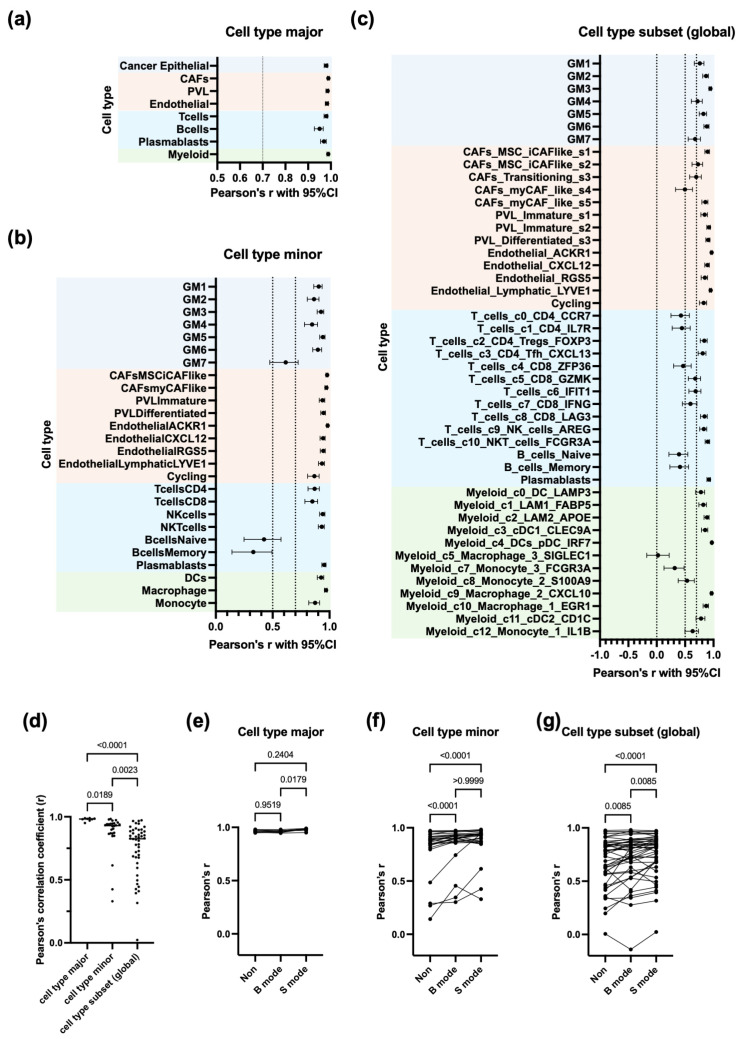
Evaluation of deconvolution accuracy using pseudo-bulk mixtures. (**a**–**c**) Pearson’s correlation coefficients (r) with 95% confidence intervals between estimated and true cell-type proportions in pseudo-bulk mixtures for the major (**a**), minor (**b**), and subset (**c**) signature matrices. Deconvolution was performed using CIBERSORTx with S-mode batch correction. (**d**) Deconvolution accuracy (Pearson’s r) compared across matrix granularities (major, minor, and subset) under S-mode batch correction. Each dot represents an individual cell type, and the horizontal bars denote the median correlation within each granularity. Statistical differences among granularities were assessed using the Kruskal–Wallis test, followed by Dunn’s multiple comparisons test. (**e**–**g**) Comparisons of deconvolution accuracy across batch correction modes (none, B-mode, and S-mode) for each matrix granularity are shown. Each dot represents an individual cell type within the corresponding signature matrix. Statistical significance was evaluated using the Friedman test, followed by Dunn’s multiple comparisons test.

Deconvolution performance was evaluated using pseudo-bulk mixtures, and accuracy was assessed using Pearson’s correlation between the estimated and true cell-type proportions under S-mode batch correction. At the major cell type level (Figure 1a), all categories showed strong correlations (r > 0.9). At the minor level (Figure 1b), most cell types exhibited r > 0.7, whereas a few cells such as naïve and memory B cells showed reduced concordance (r < 0.5). At the subset level (Figure 1c), the correlations were more variable; seven subsets (CAFs_myCAF_like_s4, T_cells_c0_CD4_CCR7, T_cells_c1_CD4_IL7R, T_cells_c4_CD8_ZFP36, B_cells_Naïve, B_cells_Memory, and Myeloid_c7_Monocyte_3_FCGR3A) showed r < 0.5. In addition, Myeloid_c5_Macrophage_3_SIGLEC1 exhibited a correlation that was not significantly different from zero. Overall, the median correlation coefficient decreased as the resolution became finer, with values of 0.98 for major types, 0.93 for minor types, and 0.83 at the subset level, respectively (Figure 1d).

A comparison of the batch correction modes (Figure 1e–g) demonstrated that S-mode generally produced the highest correlations across the resolution levels. Although not all differences reached statistical significance, S-mode consistently outperformed B-mode and the uncorrected condition.

Spearman’s rank correlations showed similar trends with slightly lower values (Supporting Information S2; Supplementary Figure S2). MAE analysis (Supporting Information S2; Supplementary Figure S3) indicated that errors were generally within ~5% for most cell types, suggesting no major systematic bias across the matrix.

### 3.2. Evaluation of Spillover Characteristics Across Matrix Conditions

**Figure 2:**
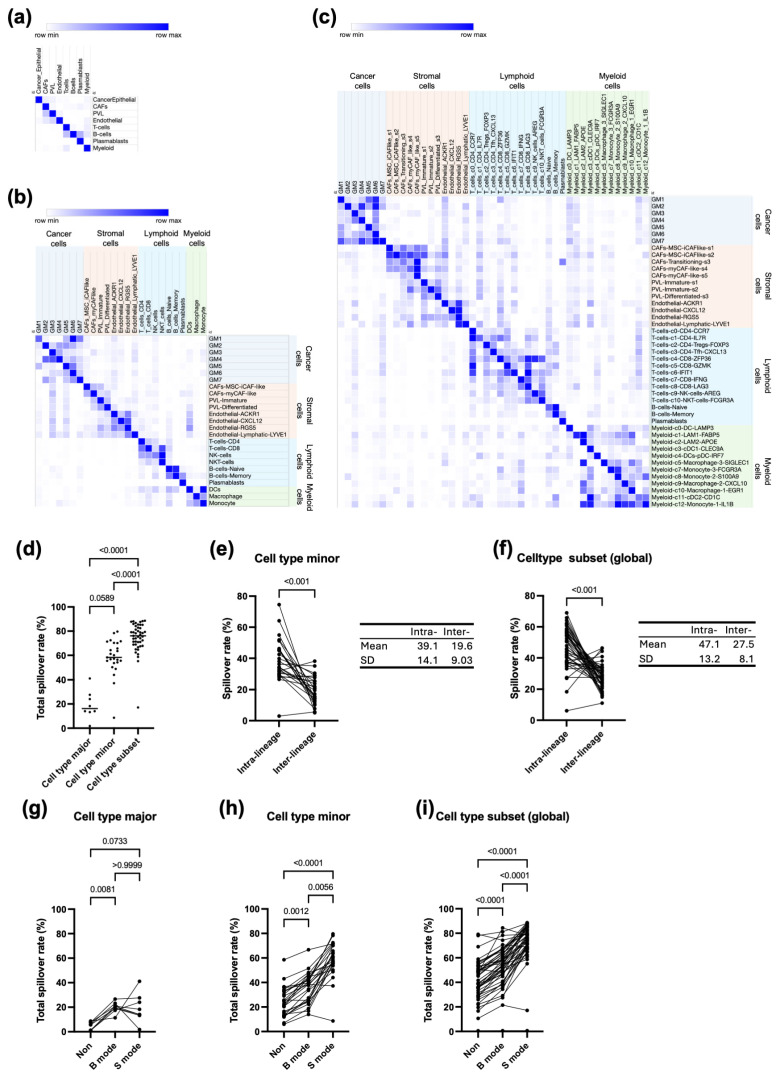
Evaluation of spillover across cell types and batch correction modes. (**a**–**c**) Spillover heatmaps generated from the deconvolution of pure cell-type samples using CIBERSORTx with S-mode batch correction, corresponding to the major (**a**), minor (**b**), and subset (**c**) signature matrix granularities. Color intensity represents the row-wise normalized values (row min–max scaling). Rows correspond to the true input cell types, and columns correspond to the predicted (estimated) cell types. (**d**) Total spillover rates compared among matrix granularities (major, minor, and subset). Each dot represents an individual cell type, and the horizontal bars denote the median total spillover rate within each granularity. Statistical differences among granularities were assessed using the Kruskal–Wallis test, followed by Dunn’s multiple comparisons test. (**e**,**f**) Comparison of intra-lineage and inter-lineage spillover rates for the minor (**e**) and subset (**f**) matrices. Intra-lineage indicates misclassification within the same lineage (cancer, stromal, lymphoid, or myeloid), whereas inter-lineage indicates cross-lineage misclassification. (**g**–**i**) Comparison of total spillover rates among batch correction modes (none, B-mode, and S-mode) at the major (**g**), minor (**h**), and subset (**i**) levels. Statistical analyses were performed using the Wilcoxon matched-pairs signed-rank test, Friedman test, or Kruskal–Wallis test as appropriate, followed by Dunn’s multiple comparisons test. The adjusted *p*-values are shown in the plots.

Spillover analysis using pure cell-type expression profiles with S-mode batch correction (Figure 2a–c) showed that the diagonal signals were the strongest, indicating that each population was predominantly assigned to its correct cell type. As the matrix granularity increased from the major to minor and subset levels, the total spillover rate also increased. Although the spillover remained relatively low at the major level, substantial spillover was observed at both the minor and subset levels (Figure 2d).

When spillover distribution was examined, most misclassification events occurred within the same lineage (cancer, stromal, lymphoid, or myeloid), whereas cross-lineage spillover was comparatively infrequent (Figure 2e,f).

When batch correction modes were compared (Figure 2g–i), spillover showed the opposite trend to correlation-based accuracy; it was lowest without batch correction, intermediate with B-mode, and highest with S-mode. Although not all pairwise differences reached statistical significance, spillover tended to increase with the extent of batch correction.

### 3.3. Lineage-Wise Deconvolution Using Subset-Level Matrices

To assess whether lineage-wise refinement improves performance, the subset-level Signature Matrix was divided into four lineage-specific matrices (cancer, stromal, lymphoid, and myeloid), and deconvolution was performed separately for each lineage using S-mode batch correction.

**Figure 3:**
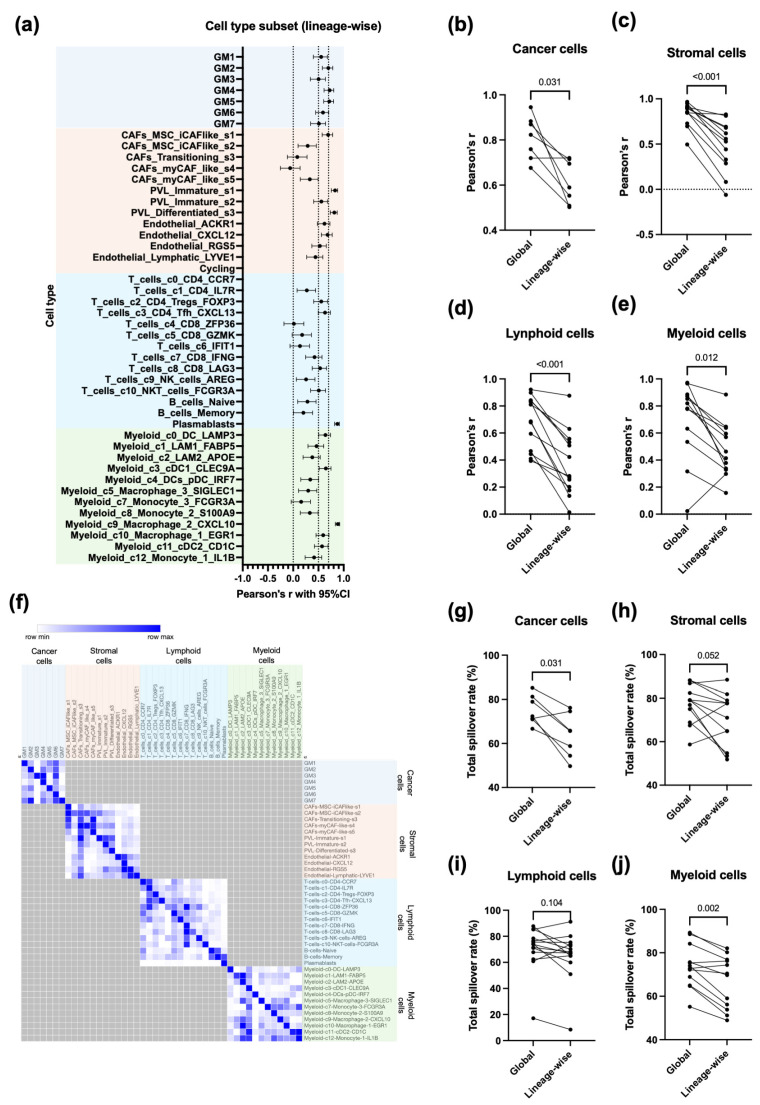
Integrated comparison of accuracy and spillover between global and lineage-wise deconvolution. (**a**) Pearson’s correlation coefficients (r) with 95% confidence intervals for estimated versus true cell-type proportions obtained using lineage-wise subset-level signature matrices (S-mode batch correction). (**b**–**e**) Paired comparisons of Pearson’s r between the global subset matrix and lineage-wise matrices for each lineage: cancer (**b**), stromal (**c**), lymphoid (**d**), and myeloid (**e**). Each point represents an individual cell type within the corresponding lineage. Statistical significance was assessed using the Wilcoxon matched-pairs signed-rank test. (**f**) Spillover heatmap generated using lineage-wise subset-level matrices with S-mode batch correction. Rows indicate the true input cell types, and the columns indicate the estimated cell types. The color intensity corresponds to row-wise normalized values (row min–max scaling), and off-lineage cells are masked in gray. (**g**–**j**) Total spillover rates for global versus lineage-wise deconvolution across cancer (**g**), stromal (**h**), lymphoid (**i**), and myeloid (**j**) lineages. Each point represents a distinct cell type within the corresponding lineage. *p*-values were calculated using the Wilcoxon matched-pairs signed-rank test.

As shown in Figure 3a, lineage-wise deconvolution consistently produced lower correlations with the ground-truth than with the global subset matrix. Paired comparisons within each lineage (Figure 3b–e) confirmed this trend.

Spillover analysis of the lineage-wise deconvolution (Figure 3f) yielded a heatmap broadly similar to the global subset analysis, with strong diagonal signals reflecting correct assignments. When the total spillover rates were compared (Figure 3g–j), lineage-wise processing resulted in a modest reduction relative to the global matrix. Although not all differences were statistically significant, cancer cells and myeloid lineages tended to show slightly lower spillover in a lineage-wise setting.

### 3.4. Validation of Cell-Type Estimates in External Bulk RNA-Seq: Composition and Marker-Based Concordance

To assess the biological plausibility of our breast-cancer-specific signature matrices, we applied the minor- and subset-level matrices to TCGA-BRCA and evaluated the resulting cell-type distributions and the concordance between inferred fractions and known marker genes, including subset-defining genes reported by Wu et al. (Supporting Information S1; Supplementary Tables S2–S4) and additional canonical lineage markers listed in the Methods, with LM22 as a comparator.

**Figure 4:**
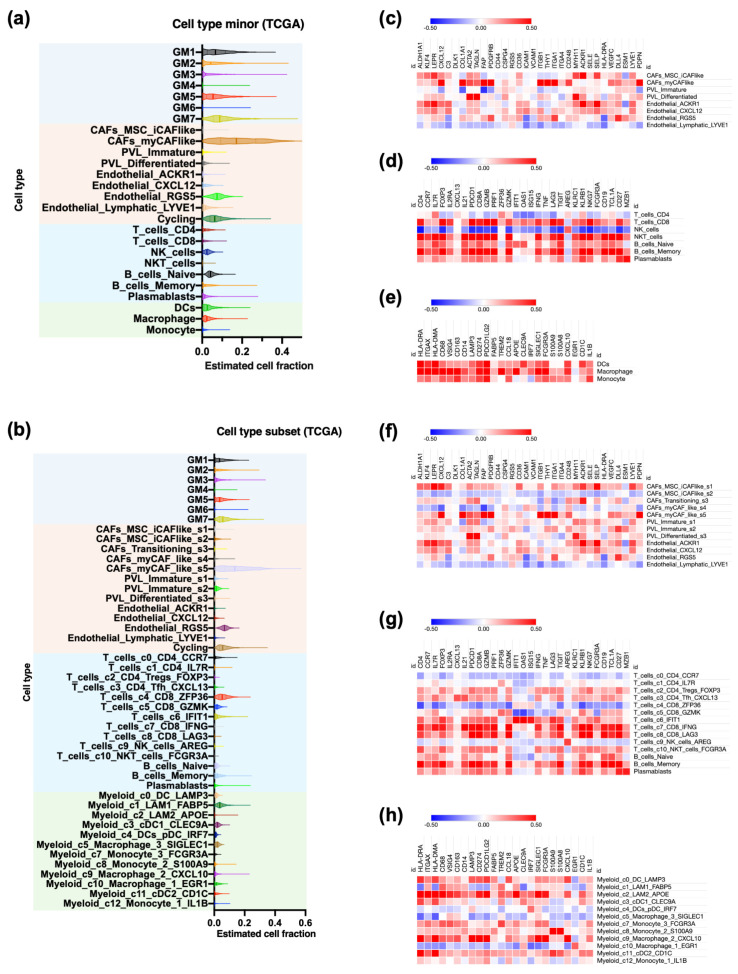
Cell-type composition and marker-gene concordance of the breast cancer-specific signature matrices in TCGA-BRCA. (**a**) Violin plots showing the estimated proportions of minor-level cell types in TCGA-BRCA bulk tumors obtained by CIBERSORTx deconvolution. (**b**–**d**) Pearson’s correlation coefficients (r) between minor-level cell-type estimates and representative marker genes for (**b**) stromal/CAF/PVL/endothelial compartments, (**c**) lymphoid compartments, and (**d**) myeloid compartments. Values are displayed on a fixed –0.50 to +0.50 scale. (**e**) Estimated proportions of subset-level cell types obtained from CIBERSORTx deconvolution. (**f**–**h**) Pearson’s correlation coefficients (r) between subset-level estimates and representative marker genes for (**f**) stromal/CAF/PVL/endothelial subsets, (**g**) lymphoid subsets, and (**h**) myeloid subsets, using the same −0.50 to +0.50 scale.

In both the minor- and subset-level results (Figure 4a,b), cancer epithelial subsets (GM1–GM7) and CAF populations consistently represented the predominant components of TCGA-BRCA, whereas other stromal and immune cell populations appeared at low-to-intermediate levels. The overall distribution of the estimated cell fractions was broadly consistent with the cellular composition typically observed in breast cancer tissues. The subset-level matrix exhibited a composition similar to that of the minor level with a wider spread of values, reflecting its finer granularity.

At the minor cell-type level (Figure 4c–e, Supporting Information S1; Supplementary Tables S8–S10), correlations with representative lineage-associated markers demonstrated that most populations exhibited patterns consistent with their expected transcriptional identities, supporting the overall biological plausibility of the deconvolution estimates. Because covariation among cell populations can occur within bulk tumors and closely related subsets naturally share portions of their marker-gene repertoires, correlations with non-specific genes are unavoidable to some extent. Nevertheless, most minor-level populations exhibited marker-concordant patterns.

However, a few minor-level groups showed discordant associations. For CD4 + T-cell populations, naïve/central-memory-associated (*CCR7*) and TH1-like effector-memory–associated (*IL7R*) markers exhibited the expected positive correlations, whereas regulatory T-cell markers (*FOXP3*, and *IL2RA*) and T follicular helper markers (*CXCL13*, *IL21*, and *PDCD1*) were negatively correlated, resulting in an inconsistent marker structure across the CD4 compartment. For natural killer (NK) cells, the canonical NK/cytotoxic markers (*NKG7*, *KLRC1*, *GZMB*, and *PRF1*) were broadly negatively correlated, whereas *AREG*, a gene known to characterize tumor-associated tissue-resident NK phenotypes in breast cancer, showed a positive association. These results suggested that the inferred NK population at the minor level reflects a tissue-resident, AREG-high NK state in breast tumor tissue, rather than a circulating canonical NK phenotype.

At the subset level, most stromal, lymphoid, and myeloid populations showed correlation patterns consistent with their expected lineage-defining markers (Figure 4f–h, Supporting Information S1; Supplementary Tables S11–S13), supporting the biological plausibility of the deconvolution results. However, several subsets exhibited clear discrepancies. In the stromal compartment, CAFs_MSC_iCAFlike_s2 showed inverse rather than positive correlations with iCAF-related markers, whereas CAFs_myCAF_like_s4 displayed clear inverse correlations with contractile/extracellular matrix-related myCAF markers, such as *ACTA2*, *TAGLN*, and *COL1A1*. Endothelial_Lymphatic_LYVE1 also showed negative correlations with lymphatic endothelial genes (*LYVE1* and *PDPN*), which was inconsistent with the expected phenotype. In the lymphoid compartment, T_cells_c0_CD4_CCR7 negatively correlated with *CCR7*, and T_cells_c4_CD8_ZFP36 showed an inverse correlation with *ZFP36*. The NK cell subset (T_cells_c9_NK_cells_AREG) exhibited broadly negative correlations with canonical NK/cytotoxic markers (*NKG7*, *KLRC1*, *GZMB*, and *PRF1*), similar to the pattern observed at the minor cell-type level. In the myeloid compartment, Myeloid_c4_DCs_pDC_IRF7 failed to show concordance with typical pDC markers other than IRF7, whereas Myeloid_c10_Macrophage_1_EGR1 correlated only with EGR1 and not with other macrophage-associated genes.

LM22, an immune-focused reference signature bundled in CIBERSORTx, was also applied to TCGA-BRCA for comparison. Because LM22 does not include non-immune lineages, its inferred immune fractions were relatively high, resulting in a distribution that differed from that of the minor cell type results (Supporting Information S2; Supplementary Figure S4a).

Supporting Information S2; Supplementary Figure S4b–c and Supporting Information S1; Supplementary Tables S14 and S15 show the correlations between LM22-derived cell type estimates and major immune marker-gene expression. Overall, both lymphoid and myeloid compartments demonstrated correlation patterns consistent with the expected lineage-defining markers. Several characteristic features were observed. The CD4 and CD8 T-cell subsets showed broadly concordant and robust correlations with representative markers, whereas plasma cells exhibited comparatively weaker associations with canonical plasma cell genes. In addition, NK cells were positively correlated with classical cytotoxic NK markers *(NKG7*, *KLRC1*, *GZMB*, and *PRF1*), a pattern that contrasts with the tumor-associated, tissue-resident NK phenotype inferred from our breast cancer-specific signature matrices.

### 3.5. Pathway-Based Functional Validation of Deconvolved Cell Populations

**Figure 5:**
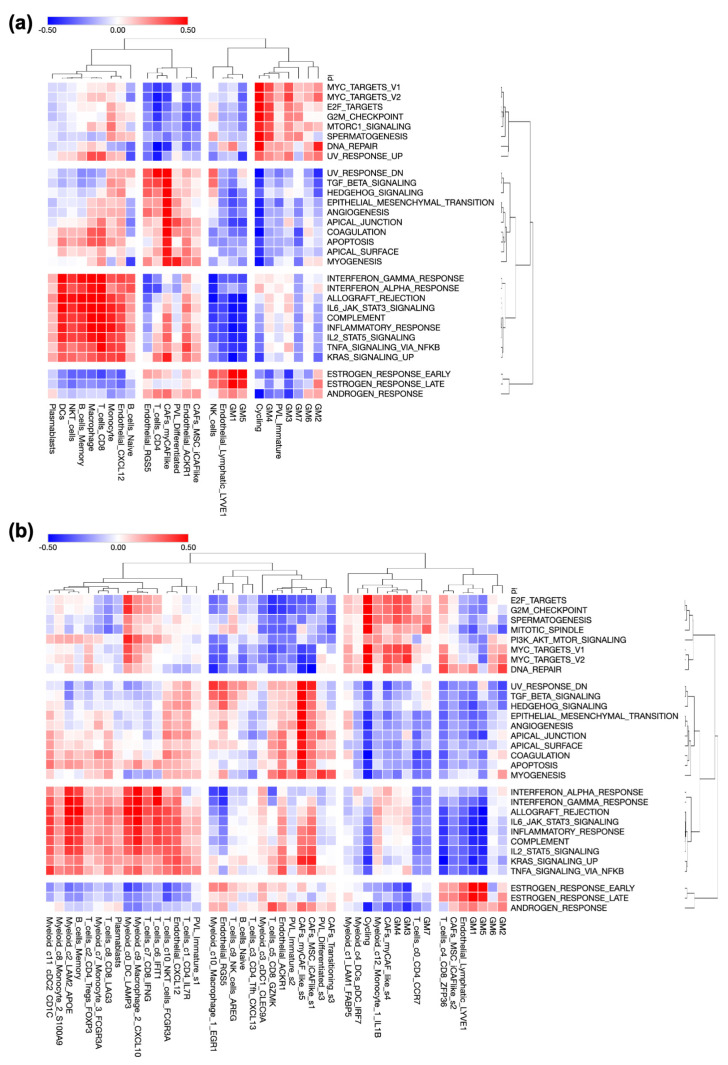
Correlation between estimated breast cancer TME cell-type fractions and Hallmark pathway activity in TCGA-BRCA. (**a**) Heatmap of Pearson’s correlation coefficients (r) between Hallmark pathway GSVA scores and minor-level cell-type fractions. (**b**) Corresponding heatmap for subset-level fractions. Correlation matrices were hierarchically clustered using one minus Pearson’s correlation with average linkage. To enhance interpretability, the 20 Hallmark pathways with the lowest maximum absolute correlations were filtered out prior to clustering. Myeloid_c5_Macrophage_3_SIGLEC1 was excluded because its estimated fractions showed a near-zero correlation with the true pseudo-bulk proportions. Filtered correlation matrices are visualized as hierarchical heatmaps. Values are displayed on a fixed −0.50 to +0.50 scale.

As shown in Figure 5a, the correlation analysis between Hallmark pathways and cell type–minor populations revealed that the pathways were segregated into four major functional clusters: proliferation and cell-cycle programs, stromal and structural programs, immune-activation pathways, and hormone-response pathways. The cell populations formed four corresponding clusters that aligned closely with these pathway-defined groups.

The proliferation-associated pathway cluster was dominated by tumor epithelial subsets, including GM2, GM3, GM4, GM6, and GM7, together with the Cycling population, reflecting the proliferative characteristics of these epithelial states. PVL_Immature was also positioned within this proliferative cluster. The stromal/structural pathway cluster was enriched for CAF populations, PVL_Differentiated, and endothelial subsets, such as Endothelial_ACKR1 and Endothelial_RGS5; T_cells_CD4 was similarly grouped within this cluster. The immune-activation pathway cluster included a broad range of immune cell populations, such as T_cells_CD8, B_cells (naïve and memory), plasmablasts, NKT_cells, dendritic cells, macrophages, and monocytes, as well as the endothelial subset Endothelial_CXCL12. Finally, the hormone-response pathway cluster comprised GM1 (luminal A) and GM5 (luminal B) in a pattern consistent with their known transcriptional identities (Supporting Information S1; Supplementary Table S1). This cluster also included NK_cells and Endothelial_Lymphatic_LYVE1.

In Figure 5b, the correlation analysis between Hallmark pathways and cell type–subset populations similarly revealed four major functional pathway clusters: hormone-response, proliferation and cell-cycle, stromal and structural, and immune-activation programs, which were organized into four corresponding groups.

The hormone-response cluster included GM1 (luminal A) and GM5 (luminal B), together with GM2 and GM6, indicating that multiple epithelial subsets were strongly linked to hormone-regulated transcriptional programs. This cluster also contained Endothelial_Lymphatic_LYVE1, CAFs_MSC_iCAFlike_s2, and T_cells_c4_CD8_ZFP36. The proliferation-associated cluster was dominated by epithelial subsets such as GM3, GM4, and GM7, along with the Cycling population, reflecting proliferative epithelial states. Several myeloid subsets, including Myeloid_c12_Monocyte_1_IL1B and Myeloid_c4_DCs_pDC_IRF7, also mapped to this cluster. The stromal/structural cluster comprised multiple CAF subsets, PVL_Differentiated, PVL_Immature_s2, and endothelial subsets, such as Endothelial_ACKR1 and Endothelial_RGS5, representing the fibroblastic and vascular stromal niches. This cluster also included several lymphoid subsets, including T_cells_c3_CD4_Tz_CXCL13 and B_cells_Naïve. The immune-activation cluster included a broad spectrum of immune populations, including: multiple CD8 T-cell subsets, CD4 T-cell subsets including Tregs, NK, and NKT populations, B-cell subsets (naïve and memory), plasmablasts, and diverse macrophage, monocyte, and dendritic cell subsets. Endothelial_CXCL12 was also positioned within this cluster, reflecting its association with immune-related signaling pathways.

For both the cell type–minor and cell type–subset analyses, full clustering results using all 50 Hallmark pathways and all deconvolved cell populations are provided in Supporting Information S2; Supplementary Figure S5a,b. A complete list of correlation coefficients and corresponding *p*-values is shown in Supporting Information S1; Supplementary Tables S16 and S17.

### 3.6. Association Between Deconvolved Cell-Type Fractions and pCR in the I-SPY2 Trial

**Figure 6:**
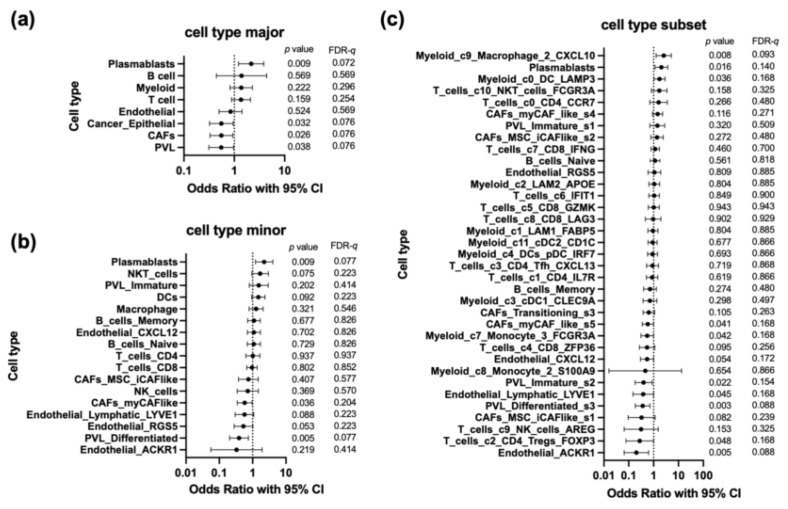
Association between deconvolved cell-type fractions and pCR in the I-SPY2 pembrolizumab + paclitaxel cohort. At the (**a**) major, (**b**) minor, and (**c**) subset levels, deconvolved cell-type fractions were tested for their association with pCR using univariable logistic regression. Odds ratios (ORs) and 95% confidence intervals (CIs) are shown for each cell population. Cell types with unstable OR/CI estimates or low deconvolution accuracy were excluded from the analysis. *p*-values and FDR-adjusted q-values are shown, and q < 0.1 was considered statistically significant.

In the I-SPY2 pembrolizumab + paclitaxel cohort, several cell populations were significantly associated with pCR across annotation levels (Figure 6a–c). At the major level, plasmablasts were positively associated with pCR, whereas CAF and PVL populations were negatively associated. At the minor level, plasmablasts were positively associated with pCR, whereas PVL_Differentiated was negatively associated. At the subset level, positive association with pCR was observed for Myeloid_c9_Macrophage_2_CXCL10, whereas negative associations were observed for PVL_Differentiated_s3 and Endothelial_ACKR1. When examined at the lineage level, plasmablast-, CAF-, and PVL-lineage populations showed similar directional trends across annotation resolutions, although statistical significance was not consistently retained after FDR correction.

## 4. Discussion

In this study, we developed multiresolution CIBERSORTx signature matrices specifically tailored to the breast cancer TME and systematically assessed their performance within this reference framework. Although previous tumor-derived matrices have been evaluated primarily at the broad immune or stromal levels, the behavior and interpretability of fine-grained breast cancer-specific subsets remain poorly defined. By integrating pseudo-bulk benchmarking, spillover profiling, lineage-wise analysis, and external validation, we identified the practical accuracy and limitations unique to this matrix, thereby clarifying its overall performance and key considerations relevant to its application.

Across the pseudo-bulk analyses, the major-, minor-, and subset-level populations were recovered with high accuracy (Figure 1a–c), demonstrating that fine-grained deconvolution within the breast cancer TME is feasible. In contrast, a limited set of immune subsets showed reduced accuracy (r < 0.5), a pattern consistent with large-scale benchmarking studies such as the DREAM Challenge [26], which similarly reported difficulty in distinguishing transcriptionally similar immune populations. In addition, the reduced-accuracy subsets included the rare populations Myeloid_c5_Macrophage_3_SIGLEC1 and Myeloid_c7_Monocyte_3_FCGR3A. The very low cell numbers of these populations in both the reference and pseudo-bulk mixtures suggest that limited sampling depth and transcriptional proximity were major contributors. Although our correlations exceeded those reported in the original study by Wu et al. (median r = 0.64), this likely reflects differences in pseudo-bulk construction. Wu et al. used smaller and more heterogeneous cell pools, whereas our 5000-cell mixtures minimized stochastic noise.

Spillover analysis showed that misclassification occurred predominantly among transcriptionally similar subsets within the same lineage, whereas cross-lineage interference was limited (Figure 2a–c). This indicates that most estimation errors arise from local ambiguity rather than the instability of the matrix as a whole. Lineage-wise deconvolution further demonstrated that restricting the reference to single lineages markedly reduced accuracy (Figure 3a–e), consistent with the loss of cross-lineage expression contrast on which ν-SVR model depends for effective separation [27,28].

Although S-mode batch correction produced the highest correlations, it also increased spillover relative to the uncorrected condition (Figure 2g–i). This likely reflects the tendency of S-mode to pull mixture profiles toward the reference distribution, thereby compressing the expression contrasts among closely related subsets. In our benchmarking framework, in which both the mixture and reference were derived from the same scRNA-seq dataset, the absence of platform- or batch-level discrepancies meant that leaving the data uncorrected preserved the original dynamic range, resulting in lower spillover. However, this behavior was specific to our validation setting, and similar results should not be assumed for typical bulk RNA-seq analyses, where substantial platform and batch differences between the mixture and reference are expected.

Taken together, the accuracy benchmarking, spillover analysis, and lineage-wise deconvolution highlight several practical considerations for the downstream use of this matrix. Practical recommendations for interpretation and application of the present breast cancer-specific multiresolution matrix are summarized in Supplementary Table S18.

Biological validation showed that most cell populations exhibited the expected associations with canonical marker genes (Figure 4c–h), indicating good overall concordance. However, several subsets displayed correlation patterns that deviated from prior expectations.

At the minor level, CD4 T cells did not fully align with the canonical marker profiles (Figure 4c), which was not unexpected, given that this population contains multiple subsets with distinct transcriptional programs. At the subset level, several groups showed discordance with the canonical markers (CAFs_MSC_iCAFlike_s2, CAFs_myCAF_like_s4, T_cells_c0_CD4_CCR7, T_cells_c4_CD8_ZFP36, T_cells_c9_NK_cells_AREG, Myeloid_c4_DCs_pDC_IRF7, and Myeloid_c10_Macrophage_1_EGR1) (Figure 4f–h). These discrepancies may, in part, reflect quantitative inverse correlations (“intra-lineage trade-offs”) among closely related subsets within the same lineage. In such configurations, the expected marker signal for a given subset can be attenuated or overridden by the relative abundance of neighboring subsets, resulting in “non-canonical” correlation patterns in bulk RNA profiles.

Therefore, we compared the correlation structures derived from the pseudo-bulk mixtures and TCGA-BRCA tumors (Supporting Information S2: Supplementary Figure S6a–d; Supporting Information S1: Supplementary Tables S19–S22). Because pseudo-bulk mixtures contain no biological covariation, the residual correlations obtained by subtracting pseudo-bulk correlations from TCGA correlations (Δr) provide a qualitative indicator of lineage–internal biological coupling. Several subsets that exhibited discordant correlations in Figure 4f–h, such as CAFs_MSC_iCAFlike_s2, CAFs_myCAF_like_s4, T_cells_c9_NK_cells_AREG, and Myeloid_c10_Macrophage_1_EGR1, also showed clear residual correlations with adjacent subsets. Although Δr does not represent a formal statistical decomposition, these findings support the possibility that some discordant marker associations reflect true biological trade-offs, rather than inaccuracies in the signature matrix. Importantly, subset-specific markers (e.g., *AREG* and *EGR1*) still showed the expected directional associations, indicating that these discrepancies do not necessarily imply a matrix failure.

In the Hallmark pathway analysis, most of the estimated cell populations showed biologically coherent associations with major tumor-related programs (Figure 5a,b), supporting the ability of our matrix to recapitulate bulk-level transcriptional states. In contrast, several fine-grained subsets displayed patterns that did not conform to canonical functional clusters. Although technical sources of variation such as intra-lineage compositional trade-offs and bulk RNA-seq-driven covariation may contribute to these discrepancies, an equally important explanation is that many of these subsets lack clearly established biological identities. Transcriptional groups, such as CD8_ZFP36, CD8_GZMK, IFIT1^+^ T cells, and LAM1/LAM2 macrophages, were originally defined as distinct single-cell states in the Wu et al. dataset; however, their immunological roles remain poorly characterized. These populations likely represent tumor- or context-specific cell states that do not fit the conventional pathway-based classifications.

Accordingly, the absence of Hallmark alignment should not be interpreted as an inconsistency, but rather as an indication that our matrix resolves biologically plausible, yet currently underdefined, states within the breast cancer TME. However, interpretations regarding these non-canonical marker and pathway associations remain exploratory and were not validated by orthogonal experimental approaches such as spatial transcriptomics or multiplex immunofluorescence. Further investigations of these subsets are required to elucidate their functional significance.

To further contextualize the subsets that were difficult to interpret based on Hallmark pathways, we examined their associations with TIDE-derived functional scores (Supporting Information S2; Supplementary Figure S7). Although the signs of these correlations cannot be directly interpreted as functional “directionality” due to lineage-internal compositional trade-offs inherent to bulk RNA-seq, they remain informative for understanding the relative positioning of closely related subsets. Despite these constraints, the cytotoxicity-related profile of the NK cell population inferred using our matrix was notably distinct from that inferred using LM22. In particular, our matrix identified an AREG-high NK cell population, consistent with the transcriptional features of tissue-resident NK cells, which differ from circulating canonical NK cells and have been reported to exhibit immunoregulatory or immunosuppressive roles [29]. Such features are difficult to capture using LM22, which is based on peripheral blood-derived reference profiles, but were clearly reflected when using a tumor-derived scRNA-seq reference. Thus, although both matrices output a population labeled “NK,” they likely represent biologically distinct cell states. This underscores the importance of using tissue-derived signature matrices and highlights the capacity of our breast tumor-specific matrix to resolve NK cell diversity within the TME.

The associations observed in this study between plasmablasts, CXCL10^+^ macrophages, CAF lineages, PVL lineages, and pCR (Figure 6a–c) are notable because they align closely with the functional mechanisms described in basic immunology and in studies of other tumor types. Plasmablast and B-cell lineages have been implicated in promoting intratumoral B-cell immunity and tertiary lymphoid structure formation [30,31,32]; CXCL10-producing macrophages provide key CXCR3 ligands required for CD8^+^ T-cell recruitment and for the efficacy of combined PD-1 and CTLA-4 blockade [33,34]; CAF- and TGF-β–driven stromal programs contribute to fibrosis and T-cell exclusion [35,36,37,38,39]; and PVL/pericyte lineages have been linked to immunosuppressive vascular niches [40]. Together, these findings suggest that the breast-cancer-specific signature matrix developed in this study captures immunobiological features of the breast cancer TME with clinical coherence and provides preliminary evidence that these cell populations may be associated with immunotherapy responsiveness in breast cancer. However, given the limited size of the I-SPY2 cohort and the exploratory nature of these analyses, further validation in larger independent cohorts will be required.

The present study has some limitations. Most importantly, all findings, including accuracy, spillover behavior, and the effects of batch correction or lineage-wise settings, reflected the characteristics of this specific breast cancer-derived signature matrix. These properties should not be assumed to generalize to matrices constructed from different scRNA-seq datasets or alternative annotation frameworks. Second, pseudo-bulk mixtures and pure samples were generated using the same scRNA-seq reference framework used for matrix construction. Although cells used for matrix construction were excluded from technical validation analyses, both datasets ultimately originated from the same limited patient cohort and annotation framework, and therefore optimistic performance estimates or cohort-specific bias cannot be completely excluded. In addition, because technical validation relied primarily on simulated pseudo-bulk mixtures rather than independent real-world bulk RNA-seq datasets with matched ground-truth cell compositions, translational reliability in clinical bulk transcriptome analyses may have been limited. This design was adopted because the fine-grained subset annotations used in the present study were specifically defined within the Wu et al. dataset. Although the Wu et al. dataset represented one of the largest publicly available breast cancer scRNA-seq resources at the time of this study, the inclusion of 26 tumors may still have been insufficient to fully capture the inter-patient heterogeneity and biological diversity across breast cancer subtypes. Third, several fine-grained non-canonical subsets showed limited marker or pathway concordance, restricting their biological interpretability in bulk analyses. These factors must be considered when applying the matrix to external datasets.

## 5. Conclusions

In this study, we constructed a breast cancer-specific CIBERSORTx signature matrix derived from tumor scRNA-seq data and performed a multi-angle evaluation to establish practical guidelines for its application. This framework may facilitate translational analyses of existing breast cancer bulk transcriptome cohorts by providing a systematically evaluated and practically interpretable reference matrix capable of resolving tumor-adapted immune and stromal cell states that are difficult to capture using conventional blood-derived reference matrices.

## Data Availability

All datasets analyzed in this study were obtained from publicly available sources (accessed on 1 May 2025). The breast cancer-specific CIBERSORTx signature matrices, associated reference files, and other processed resources generated during this study are publicly available in Zenodo at https://doi.org/10.5281/zenodo.17788679.

## Supplementary Materials

The following supporting information can be downloaded at: https://www.mdpi.com/article/10.3390/mps9030088/s1, Supporting Information S1 (Supporting_information_1.xlsx). File format: .xlsx Title: Supplementary Tables S1–S22. Description: This Excel file contains 15 worksheets comprising Supplementary Tables S1–S22 The tables include: summary annotations and inferred functional characteristics of GM1–GM7 cancer epithelial cells and fine-grained subsets (Tables S1–S4); cell counts for all major, minor, and subset categories used in the construction of the signature matrices (Tables S5–S7); correlation analyses between deconvolved cell-type fractions and representative marker genes in TCGA-BRCA (Tables S8–S13); comparative analyses using the LM22 signature matrix, including marker-based correlations (Tables S14 and S15); complete Pearson correlation coefficients and associated *p*-values for correlations between Hallmark GSVA scores and estimated cell-type fractions (Tables S16 and S17); practical guidelines for interpretation and application of the breast cancer-specific multiresolution CIBERSORTx matrix (Table S18); and full correlation matrices of true cell-type population proportions used to generate pseudo-bulk mixtures (Table S19), together with corresponding correlation matrices derived from deconvolution results and residual correlations (Δr) (Tables S20–S22). Supporting Information S2 (Supporting_information_2.pptx). File format: pptx. Title: Supplementary Figures S1–S7. Description: This PowerPoint file contains Supplementary Figures S1–S7 supporting benchmarking and external validation analyses. The figures include distributions of true pseudo-bulk cell-type fractions (Supplementary Figure S1); accuracy validation based on Spearman correlation coefficients (Supplementary Figure S2); MAE-based performance evaluation (Supplementary Figure S3); comparative analyses with the LM22 signature matrix (Supplementary Figure S4); correlation heatmaps between Hallmark GSVA scores and estimated cell-type fractions (Supplementary Figure S5); correlation structures in pseudo-bulk and TCGA datasets together with residual correlations (Δr) (Supplementary Figure S6); and correlations between TIDE-derived indices, CYT, and estimated cell-type fractions (Supplementary Figure S7).

## Abbreviations

GSVA	Gene Set Variation Analysis
MAE	mean absolute error
NK	natural killer
pCR	pathological complete response
PVL	perivascular-like
RNA-seq	RNA sequencing
scRNA-seq	single-cell RNA sequencing
TCGA-BRCA	The Cancer Genome Atlas Breast Invasive Carcinoma
TME	tumor microenvironment
TPM	transcripts per million
