# Supplementary figures and images for: A Multiresolution Breast Cancer CIBERSORTx Resource Validated for Accuracy, Interpretive Limits, and Biological and Clinical Coherence in Tumor Microenvironment Deconvolution
Source: Methods Protoc. 2026 Jun 2;9(3):88. doi: 10.3390/mps9030088 (PMC13304752; doi:10.3390/mps9030088)

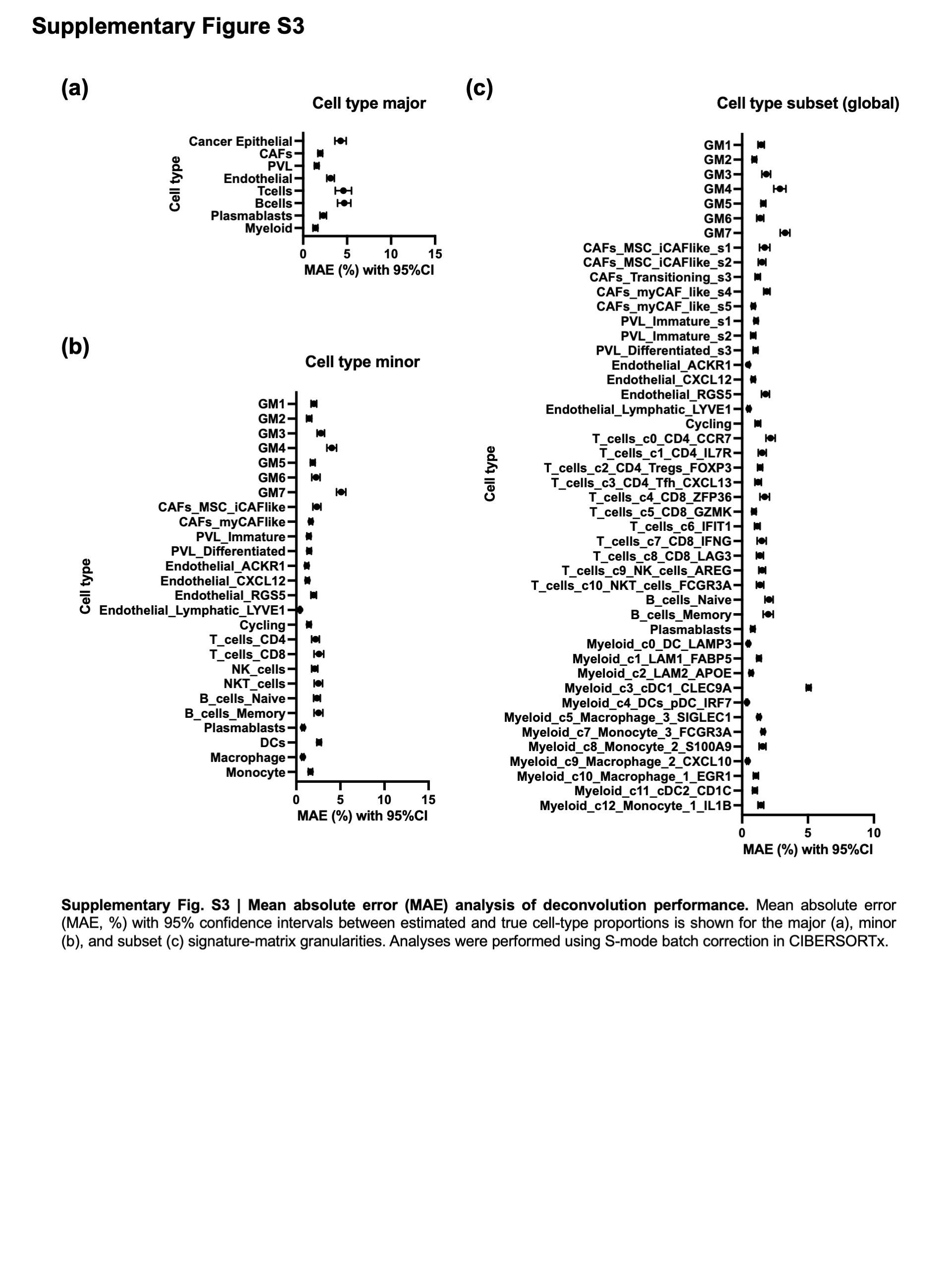

Supplement: Supplementary file 1 [file mps-09-00088-s001.zip › Supplementary files/Supporting_information_2/Supplementary Figure S3..png]

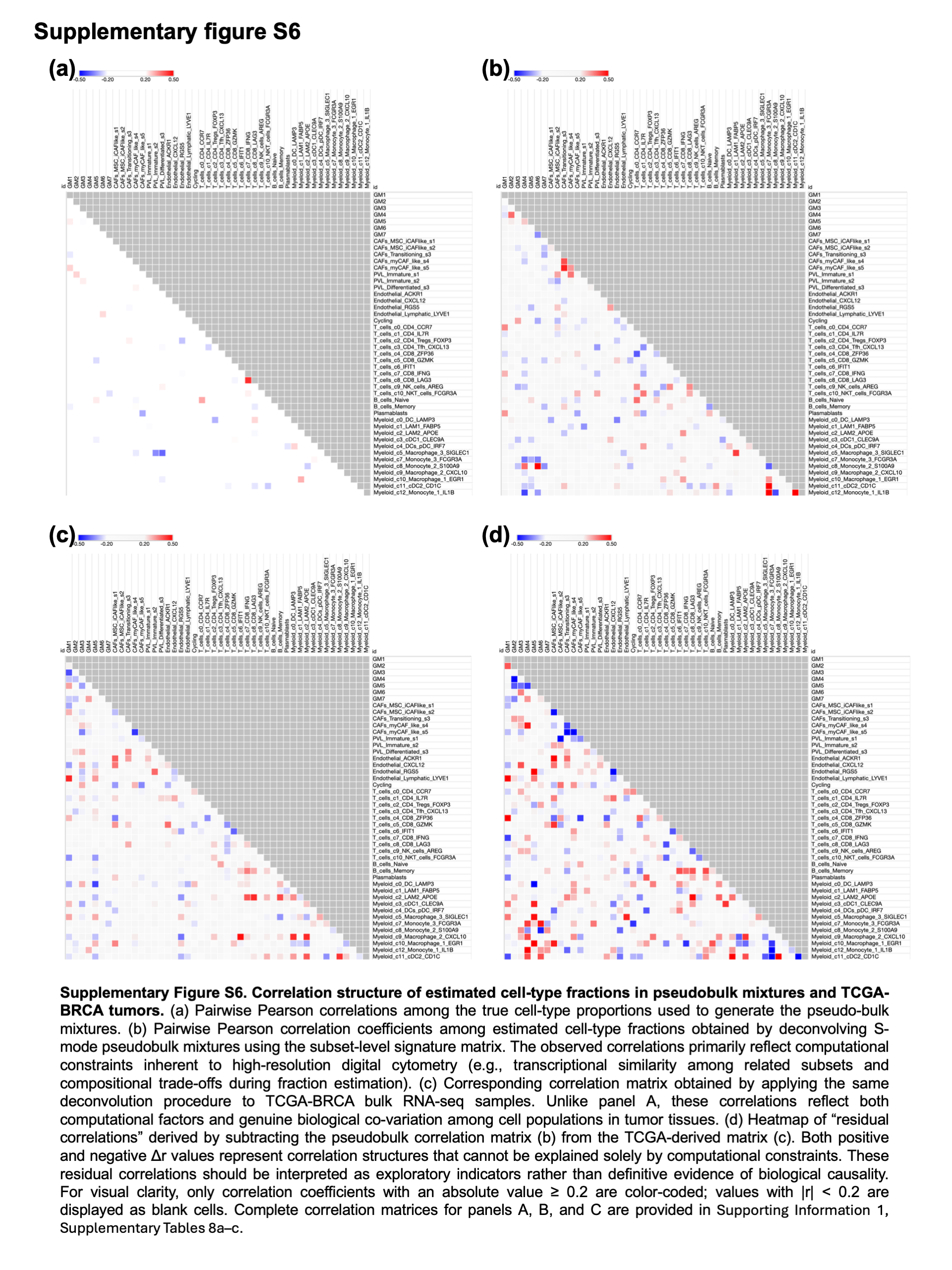

Supplement: Supplementary file 1 [file mps-09-00088-s001.zip › Supplementary files/Supporting_information_2/Supplementary Figure S6.png]

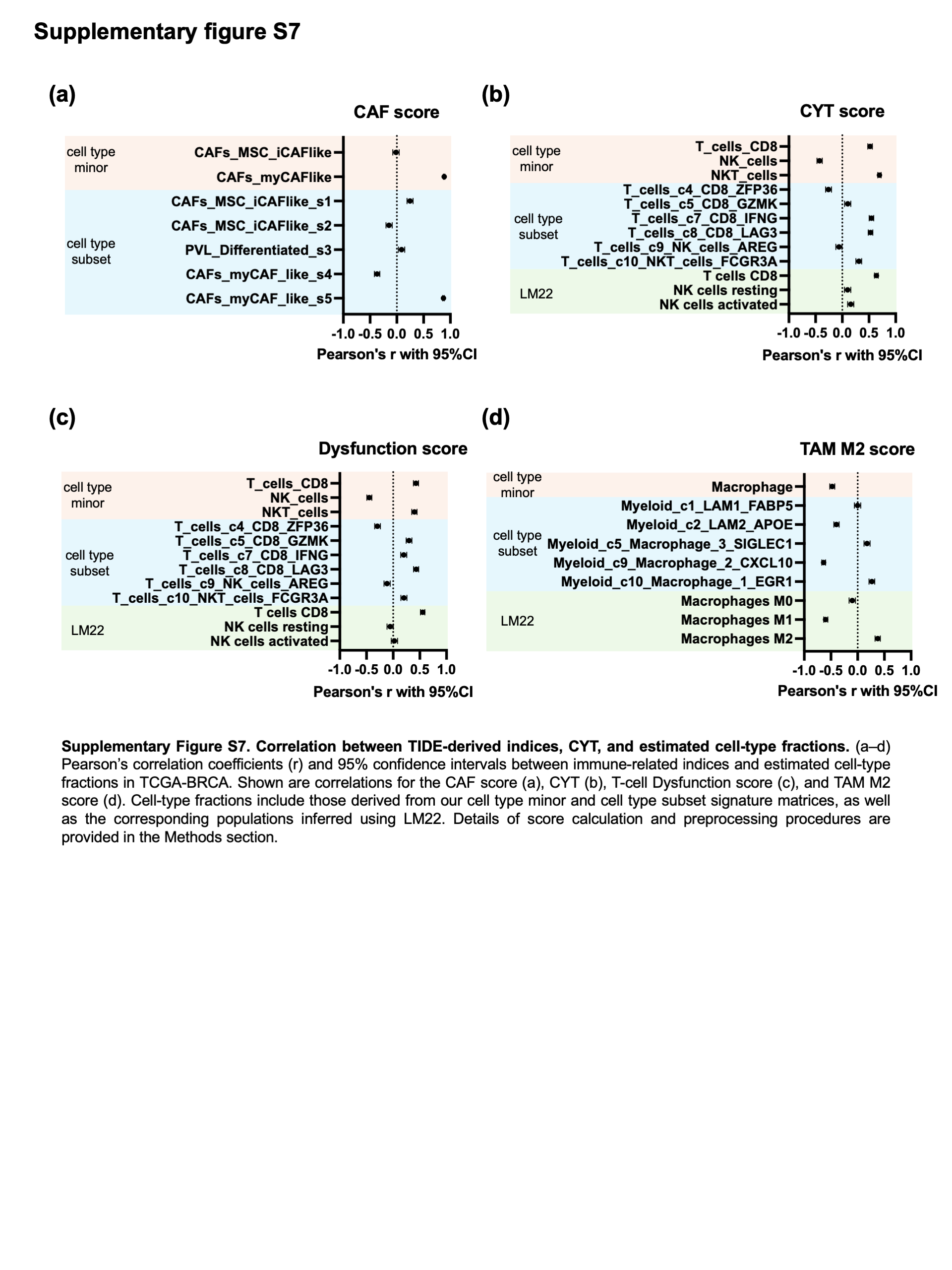

Supplement: Supplementary file 1 [file mps-09-00088-s001.zip › Supplementary files/Supporting_information_2/Supplementary Figure S7.png]

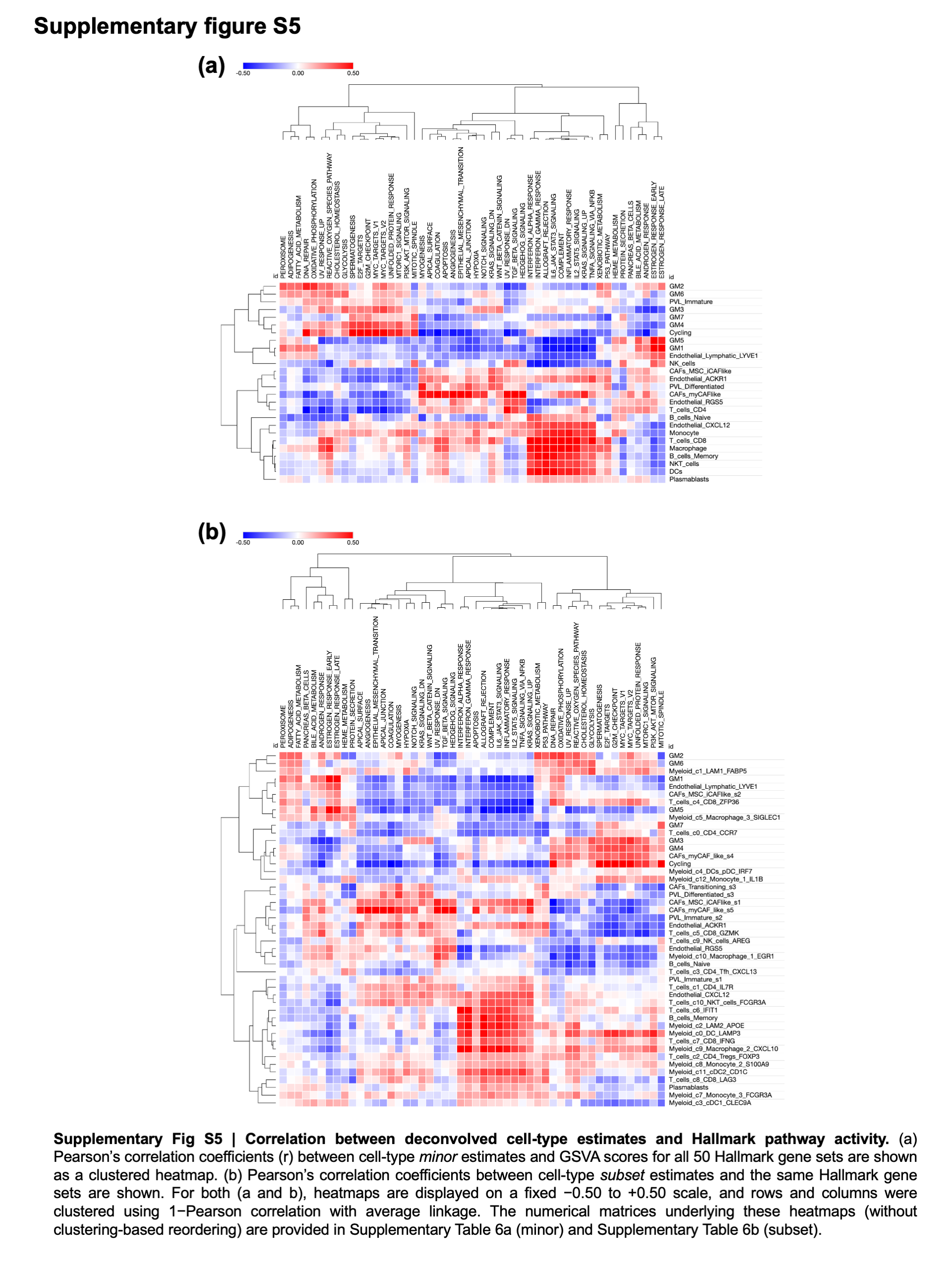

Supplement: Supplementary file 1 [file mps-09-00088-s001.zip › Supplementary files/Supporting_information_2/Supplementary Figure S5.png]

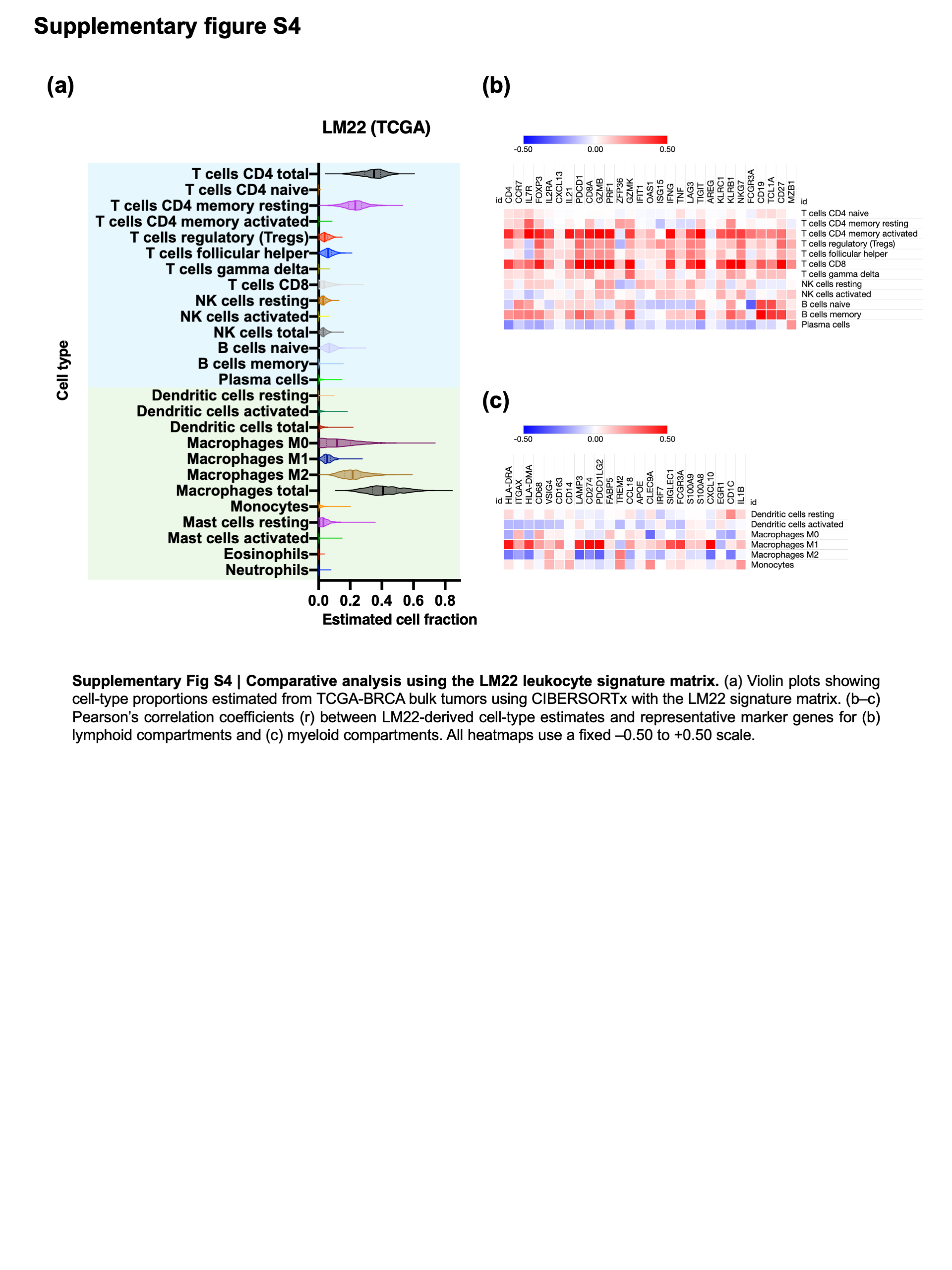

Supplement: Supplementary file 1 [file mps-09-00088-s001.zip › Supplementary files/Supporting_information_2/Supplementary Figure S4.png]

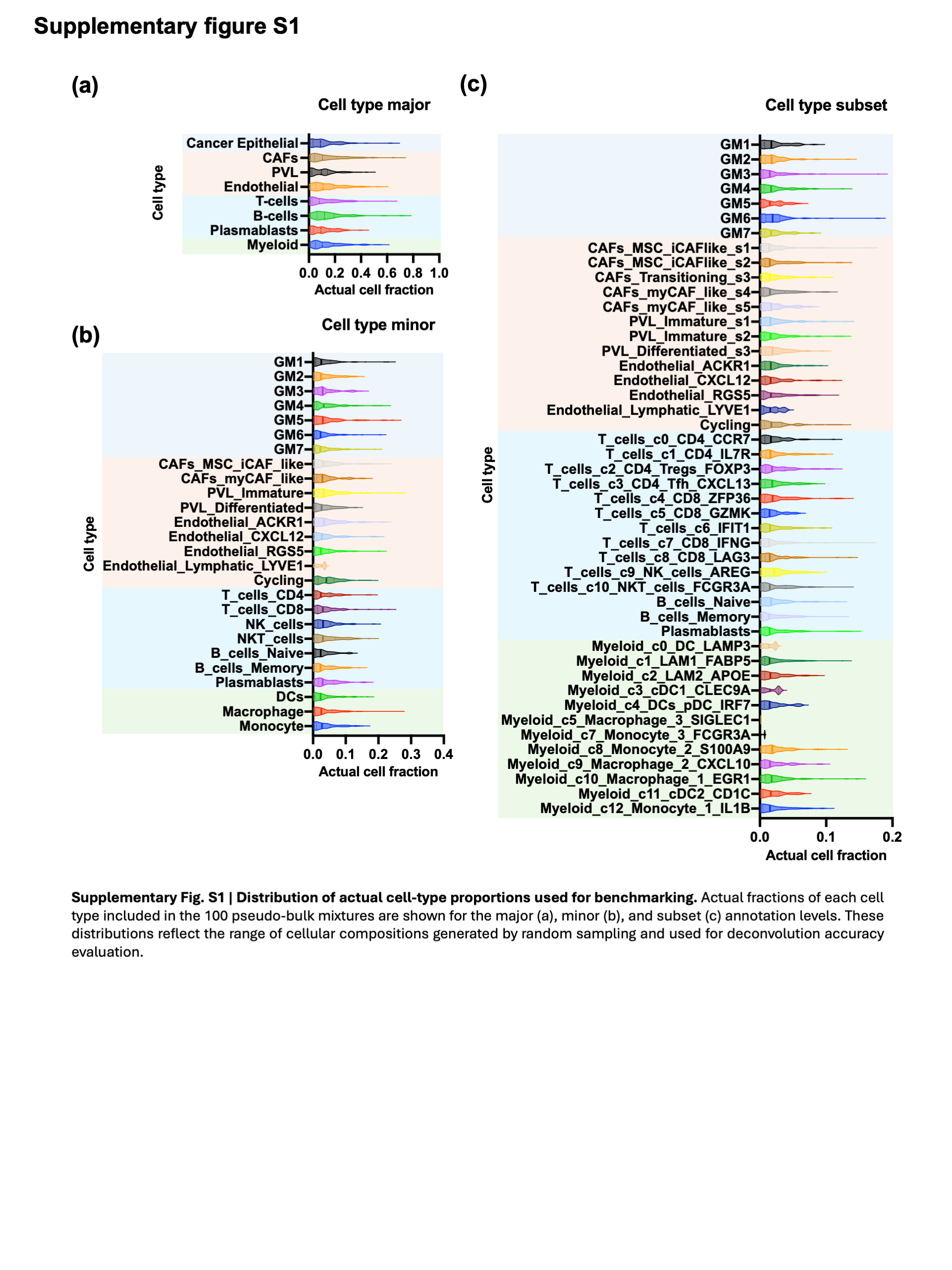

Supplement: Supplementary file 1 [file mps-09-00088-s001.zip › Supplementary files/Supporting_information_2/Supplementary Figure S1.png]

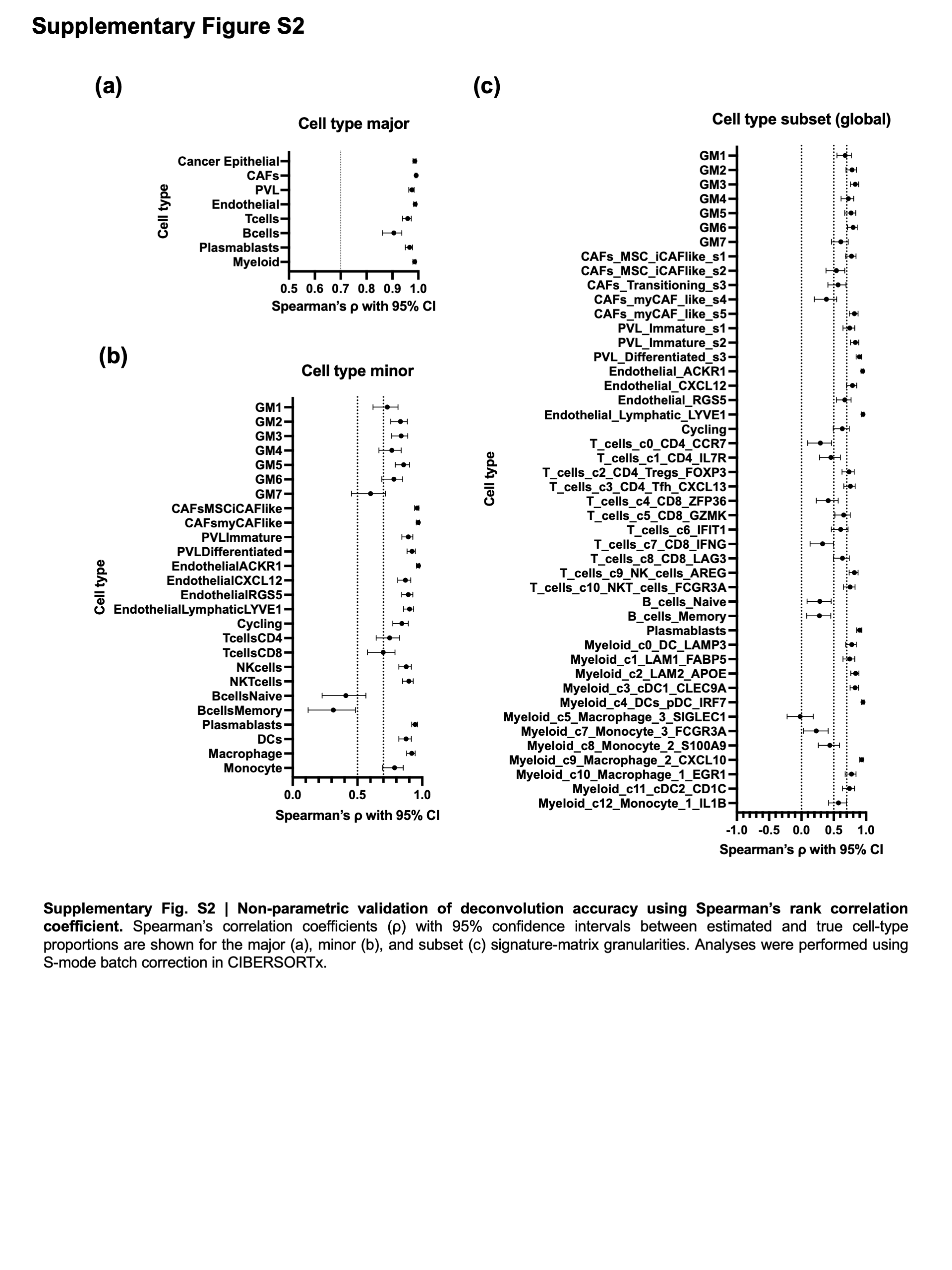

Supplement: Supplementary file 1 [file mps-09-00088-s001.zip › Supplementary files/Supporting_information_2/Supplementary Figure S2.png]
